# Combined Proteomic and Metabolomic Analysis Reveals Comprehensive Regulation of Somatostatin DNA Vaccine in Goats

**DOI:** 10.3390/ijms25136888

**Published:** 2024-06-23

**Authors:** Ge Qin, Li Zhang, Jiaxue Guo, Shiyong Fang, Guangxin E, Yan Zeng, Yongfu Huang, Yanguo Han

**Affiliations:** Chongqing Key Laboratory of Forage & Herbivore, Chongqing Engineering Research Centre for Herbivores Resource Protection and Utilization, College of Animal Science and Technology, Southwest University, Chongqing 400715, China; qinge0163@163.com (G.Q.); zhangli510704@163.com (L.Z.); guojiaxue1005@163.com (J.G.); fsy2022swu@163.com (S.F.); eguangxin@126.com (G.E.); zengyan@swu.edu.cn (Y.Z.); h67738337@swu.edu.cn (Y.H.)

**Keywords:** somatostatin, DNA vaccine, dazu black goats, proteomics, metabolomics

## Abstract

Somatostatin (SS) plays crucial regulatory roles in animal growth and reproduction by affecting the synthesis and secretion of growth hormone (GH). However, the mechanism by which SS regulates growth and development in goats is still unclear. In order to investigate the regulatory networks of the hypothalamus and pituitary in goats affected by *SS* DNA vaccines, in this study, we used a previously established oral attenuated *Salmonella typhimurium SS* DNA vaccine, *X9241* (ptCS/2SS-asd), to treat wethers. We analyzed the protein changes in hypothalamic and pituitary tissues using a TMT-based proteomics approach. Additionally, we examined the metabolic profiles of the serum of control and immunized wethers through untargeted metabolomics using liquid chromatography–mass spectrometry (LC–MS). Key signaling pathways were identified based on differentially expressed metabolites (DEMs) and differentially expressed proteins (DEPs). Furthermore, the effect of critical DEPs on signaling pathways was confirmed through Western blotting (WB) experiments, which elucidated the mechanism of active *SS* immunization in wethers. A proteomics analysis revealed that the expression of 58 proteins in the hypothalamus and 124 in the pituitary gland was significantly altered following *SS* vaccine treatment (fold change > 1.2 or < 0.83, *p* < 0.05). In the hypothalamus, many DEPs were associated with gene ontology (GO) terms related to neuronal signaling. In contrast, most DEPs were associated with metabolic pathways. In the pituitary gland, the DEPs were largely related to immune and nutrient metabolism functions, with significant enrichment in KEGG pathways, particularly those involving the metabolic pathway, sphingolipid signaling, and the cGMP-PKG signaling pathway. A metabolomic analysis further showed that active *SS* immunization in wethers led to significant alterations in seven serum metabolites. Notably, the sphingolipid signaling pathway, secondary bile acid synthesis, sphingolipid metabolism, and lysine synthesis were significantly disrupted. *SS* vaccines induced marked changes in hypothalamic–pituitary proteins in wethers, facilitating alterations in their growth processes. This study not only provides insights into the mechanism of the *SS* gene in regulating GH secretion in wethers but also establishes a basis for hormone immunoregulation technology to enhance livestock production performance.

## 1. Introduction

Multiple factors affect goat growth, including breed, nutrition, endocrine levels, and the environment. Among these factors, endocrine levels play a crucial role [1]. Growth hormone (GH) is secreted by the pituitary gland and exerts a comprehensive growth-promoting effect by mediating insulin-like growth factor 1 (IGF-1). GH secretion is regulated by both somatostatin (SS) and growth hormone-releasing hormone (GHRH) [2]. The development of hormone-based gene vaccines aimed at producing specific antibodies to neutralize targeted hormone concentrations in animals has led to the increasing use of these vaccines to enhance animal growth and reproductive performance. Various hormone-based gene vaccines, such as SS [3], inhibin [4], and kisspeptin-54 [5], have been reported in this field.

SS is a peptide hormone composed of 14 residues and a disulfide bond. It is primarily secreted by the hypothalamus but can also be produced by pancreatic D-cells, the stomach, and the intestines. SS inhibits the secretion of hormones associated with the digestive system, including GH, insulin, glucagon, ghrelin, and secretin [6]. A novel oral *SS-14* gene vaccine has been developed to immunize lambs, stimulating the production of SS antibodies that neutralize SS in vivo and promote animal growth [3,7]. However, the specific mechanism by which this vaccine regulates GH secretion and growth performance remains unclear.

Cumulative reports suggest that SS has different regulatory effects on pituitary GH secretion depending on the somatostatin receptors (SSTRs) to which it binds [8]. The activation of SSTRs regulates cyclic adenosine monophosphate (cAMP), inositol phosphate production, and the activity of K^+^ and Ca^2+^ channels [8]. Overexpression of SSTR2 in a cellular subclone of GHs indicates that SSTR2 is constitutively active and, through deacetylation, inhibits cAMP accumulation and rat GH promoter activity, ultimately suppressing GH synthesis [9]. Reduced cAMP levels many result in the inhibition of hormone secretion, decreased cell proliferation, and regulation of ion channels [10]. Mitogen-activated protein kinases (MAPKs) play crucial roles in signal transduction following SSTR activation. The activation of SSTRs can lead to the activation of MAPK pathways, such as extracellular signal-regulated kinase (ERK) and c-Jun N-terminal kinase (JNK) [11]. These signaling pathways can influence various cellular processes, including proliferation, differentiation, apoptosis, and metabolism.

In the central nervous system, SS functions as a neurotransmitter, facilitating communication between neurons. Additionally, its transfer from the hypothalamus to the pituitary qualifies it as a neurohormone that participates in neuroendocrine regulation [12]. However, our understanding of how signals from altered hormone levels in peripheral tissues feedback to hypothalamic neurons to affect SS hormone secretion remains limited.

The analysis of metabolomics and proteomics through multiomics approaches allows for a comprehensive study of the interactions and associations between metabolites and proteins. From a complete and multidimensional perspective, this integrated approach enables a better understanding of the integrated responses within the animal organism. This approach has been widely utilized in various studies in the field of life sciences [13]. In our laboratory, a novel oral *SS-14* gene vaccine, *X9241* (ptCS/2SS-asd), which was fused with a *CpG* adjuvant and *tPA* signaling peptide was successfully constructed in the previous period [7]. This vaccine was delivered by an attenuated *Salmonella typhimurium X9241* vector. In order to better describe the protein and metabolite regulatory networks in the hypothalamus and pituitary as well as in peripheral serum following neutralization of SS by peripheral production of SS antibodies in goats. We analyzed the changes in metabolites in the peripheral blood serum of wethers using liquid chromatography–mass spectrometry (LC–MS)-based nontargeted metabolomics. Furthermore, we analyzed the changes in proteins in the hypothalamus and pituitary tissues using TMT-based proteomic methods. Key signaling pathways were studied based on differentially abundant metabolites (DEMs) and differentially expressed proteins (DEPs). Western blotting (WB) was applied to validate the results obtained from the multiomics analysis.

## 2. Results

### 2.1. Effect of SS Vaccine Treatment on the Hypothalamic–Pituitary Proteome of Wethers

**Figure 1:**
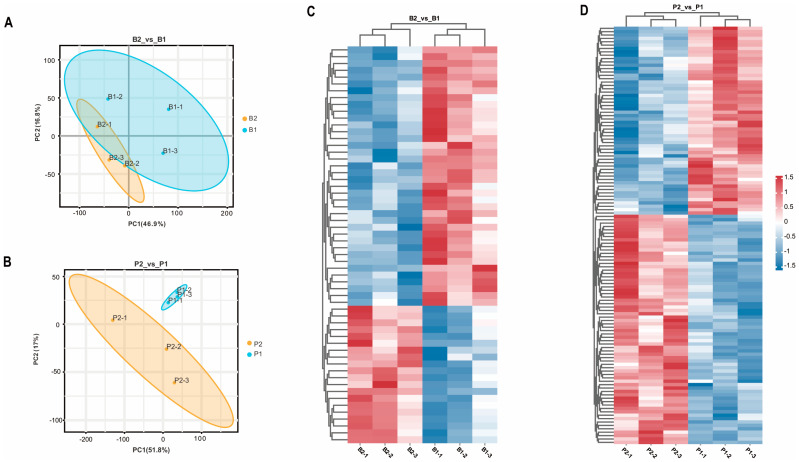
Results of principal component analysis of identified proteins in the proteome and heat map of DEPs in each treatment group. (**A**,**B**) show the PCA of proteins identified in each sample of hypothalamus (B2_vs_B1) and pituitary gland (P2_vs_P1) from goats in the immunized group and negative control group, respectively. B2-1, B2-2, and B2-3 represent hypothalamus of 3 goats in the immunized group; B1-1, B1-2, and B1-3 represent hypothalamus of 3 goats in the negative control group; P2-1, P2-2, P2-3 and P1-1, P1-2, P1-3 represent pituitary glands of three goats in the immunized and negative control groups, respectively. (**C**,**D**) show the heatmap analysis of the two groups of screened differential proteins, B2_vs_B1 and P2_vs_P1, respectively, with red and blue color indicating up- and down-regulated proteins, respectively.

To investigate the impact of SS antibodies produced in the peripheral blood of goats after oral administration of the SS-14 DNA vaccine *X9241* (ptCS/2SS-asd) on the hypothalamus and pituitary, samples from the hypothalamus (B2_vs_B1) and pituitary (P2_vs_P1) of the immunized group and negative control group were analyzed using TMT-labeled proteomics. The PCA results demonstrated significant differences in protein expression in the hypothalamus and pituitary tissues among the different treatment groups (Figure 1A,B). Within the hypothalamic and pituitary groups, 58 and 124 proteins, respectively, exhibited significant changes in expression under the influence of the SS vaccine. In the B2_vs_B1 group, 20 proteins were upregulated, and 38 proteins were downregulated (Figure 1C). Similarly, 68 upregulated proteins and 56 downregulated proteins were detected in the P2_vs_P1 group (Figure 1D).

**Figure 2:**
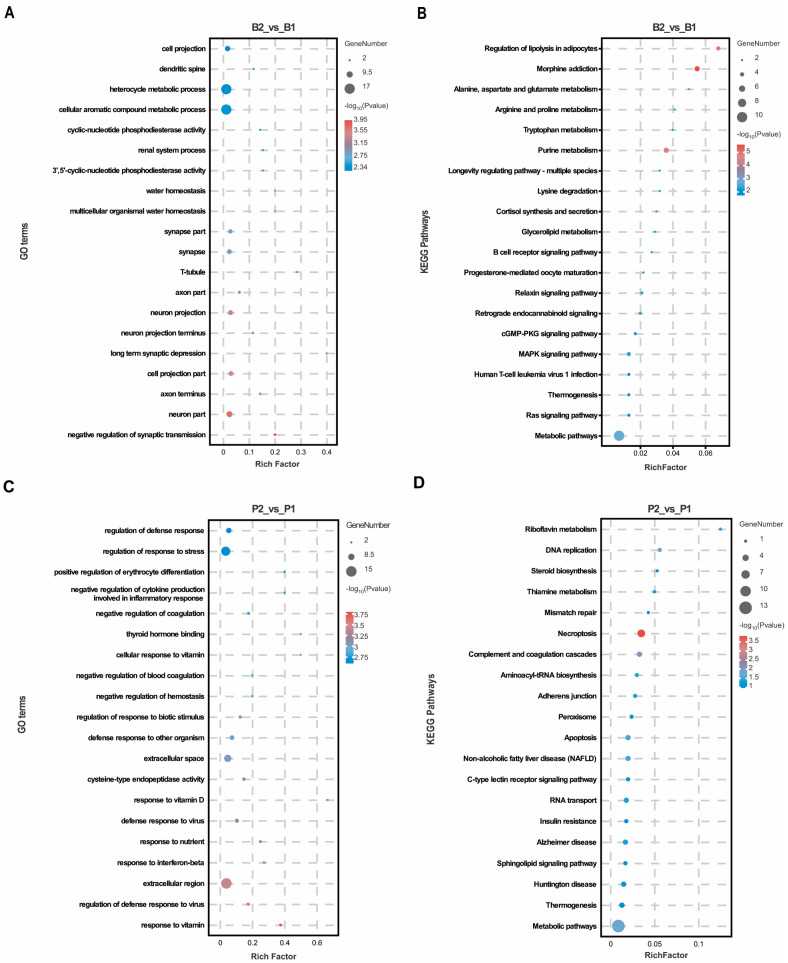
GO and KEGG enrichment analysis in hypothalamus and pituitary group proteomics. (**A**,**B**) The top 20 GO entries and KEGG signaling pathways based on the differentially expressed proteins enriched to the B2_vs_B1 group. (**C**,**D**) The top 20 GO entries and KEGG pathways based on the differentially expressed proteins enriched to the P2_vs_P1 group.

To predict the biological functions of these DEPs, GO annotation was performed separately for the hypothalamus and pituitary gland. In the hypothalamus, DEPs were significantly enriched in 329 GO terms, with a majority (77.51%) related to biological processes. The top 20 GO terms, ranked by *p*-value, were primarily associated with neuronal signaling and such as “neuron part”, “axon terminus”, “cell projection part”, “neuron projection”, “axon part”, and “synapse part”. Furthermore, the negative regulation of synaptic transmission and long-term synaptic depression were observed under the biological process category, along with cellular aromatic compound metabolic processes (Figure 2A), suggesting that the SS vaccine obstructed signaling between hypothalamic neurons. In the pituitary gland, DEPs were significantly enriched in 408 GO terms, 76.96% of which were associated with biological process terms. These functional classifications were predominantly related to immunity and nutrient metabolism, including responses to vitamins, regulation of defense response to viruses, response to interferon-beta, response to nutrients, defense response to viruses, and response to vitamin D (Figure 2C). This indicates the activation of regions responsible for growth and immunity regulation in pituitary tissue following SS vaccine treatment. Additionally, KEGG analysis revealed that DEPs in the hypothalamus were enriched mainly in metabolic pathways such as purine metabolism, lysine degradation and alanine, aspartate, and glutamate metabolism (Figure 2B). Notably, the cGMP-PKG signaling pathway, Ras signaling pathway, and MAPK signaling pathway were also significantly enriched as these pathways are crucial for regulating peptide–cell membrane receptor intracellular signaling. Similarly, KEGG pathways significantly enriched in the pituitary were associated with metabolic and signaling pathways, including thiamine metabolism and the C-type lectin receptor signaling pathway (Figure 2D).

**Figure 3:**
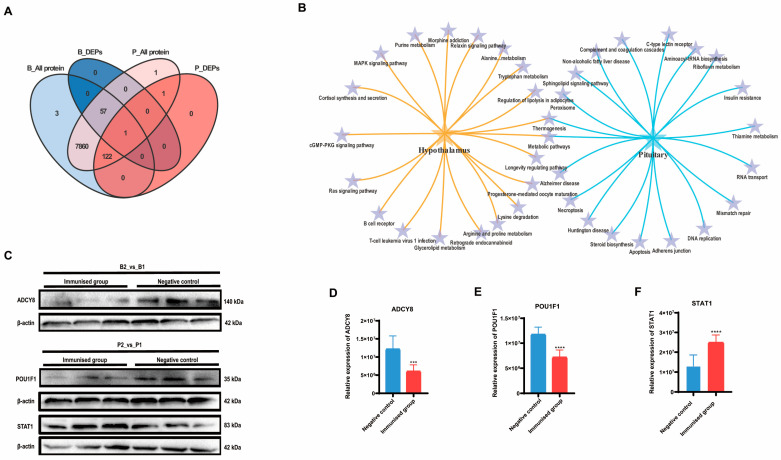
Combined analysis and validation of hypothalamus and pituitary group proteomics. (**A**) Venn plot based on the differential proteins identified in B2_vs_B1 and P2_vs_P1. (**B**) Dynamic Venn plot based on the top 20 KEGG signaling pathways enriched in the hypothalamus and pituitary, respectively. (**C**–**F**) Western blot validation of the proteomics results for both hypothalamus and pituitary groups and the relative quantification results, respectively. Error lines indicate standard deviation (*** *p* < 0.001, **** *p* < 0.0001).

A Venn analysis revealed that the only differentially expressed protein significantly affected by SS immunity in both hypothalamic and pituitary tissues was LOC102183616, ubiquinone-cytochrome c oxidoreductase. However, among the proteins identified in the pituitary tissue, all of the differentially expressed proteins identified in the hypothalamus were also present. This finding suggested a close association between the hypothalamus and pituitary (Figure 3A). Both KEGG enrichment analyses indicated the co-enrichment of thermogenic and metabolic pathways in both the hypothalamus and pituitary (Figure 3B).

We validated the expression of the crucial DEPs (Figure 3C). Adenylate cyclase type 8 (ADCY8), which is responsible for catalyzing the formation of cAMP in the hypothalamus exhibited significantly lower relative expression in the immunized group than in the negative control group (*p* < 0.05) (Figure 3D). Pituitary-specific positive transcription factor 1 (POU1F1), which plays a role in the development of the pituitary gland and hormone expression, exhibited significantly lower relative expression in the pituitary gland of the immunized group than in that of the negative control group (*p* < 0.01) (Figure 3E). In contrast, the relative expression of signal transducer and activator of transcription (STAT1) was markedly greater in the pituitary glands of the immunized group than in those of the negative control group (*p* < 0.01) (Figure 3F). These findings align with the results of the proteomic analysis (Supplementary Data S1).

### 2.2. Integrated Metabolomics and Proteomics Analysis of Regulatory Networks in SS Vaccine-Treated Wethers

**Figure 4:**
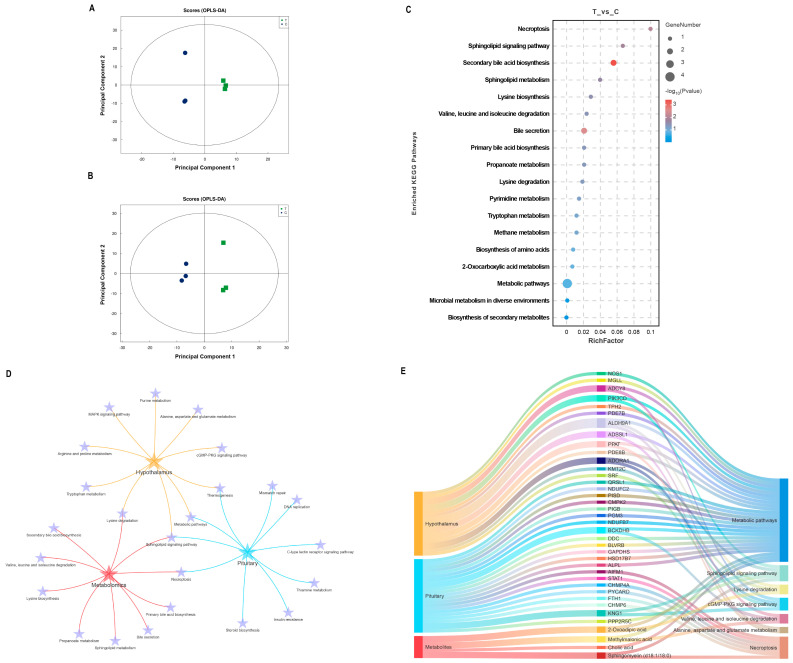
Integrated analysis of metabolomics and proteomics. (**A**,**B**) OPLS-DA score plots for the positive and negative ion modes, respectively. (**C**) Bubble diagram of KEGG pathways enriched for significantly different metabolites. (**D**) Interaction analysis of common KEGG pathways and amino acid metabolism-related pathways enriched for DEPs in the hypothalamus and pituitary and DEMs in serum. (**E**) Mulberry diagram of key KEGG pathways in hypothalamic and pituitary proteomics and serum metabolomics.

Structural characterization of metabolites in biological samples led to the identification of 135 positively charged metabolites and 140 negatively charged metabolites (Supplementary Data S2). The OPLS-DA results (Figure 4A,B) demonstrated a high consistency in metabolite expression patterns between individuals within the group, with a clear separation observed between the immunized and negative control groups. The metabolites were screened based on the criteria of FC > 1.2 or FC < 0.83, VIP > 1, and *p* < 0.05. Detailed information on the significant differences in metabolite analyses in both modes is shown in Table S2. Only two DEMs were identified in the positive ion mode, whereas six DEMs were identified in the negative ion mode. KEGG enrichment analysis of the significantly different metabolites revealed that the sphingolipid signaling pathway, secondary bile acid synthesis pathway, sphingolipid metabolism, and lysine synthesis were affected during the immune response to the SS gene vaccine in goats, as depicted in Figure 4C. Notably, DEPs in the hypothalamus and pituitary, as well as DEMs in the serum, were significantly enriched in the sphingolipid signaling pathway (Figure 4D). A comprehensive analysis of the common KEGG pathways enriched for differential proteins and metabolites, including pathways related to amino acid metabolism, is presented in Figure 4E. It is evident that the metabolic pathways and sphingolipid signaling pathways undergo the most pronounced changes. In particular, ADCY8 in the hypothalamus, which is responsible for catalyzing cAMP production, KNG1 in the pituitary gland, and serum sphingolipids (d18:1/18:0) all play crucial roles in the sphingolipid signal pathway.

## 3. Discussion

Previous studies have reported that both active and passive immunization against SS can induce an increase in GH, thereby improving growth or lactation performance in animals such as mice [14], goats [3], and piglets [15]. *SS* gene vaccines are endocytosed by antigen-recognizing cells of the intestinal mucosal immune system after they are inhaled from the mouth and nose by animals. The antigen is then presented to surrounding B cells and T cells that differentiate into plasma cells, which produce antibodies against the *SS* gene vaccine. SS antibodies are secreted and circulate freely in the blood and lymphatic circulatory system, where they bind to SS and relieve its inhibitory effect on GH [16]. However, these studies do not provide insights into whether SS antibodies can cross the blood–brain barrier or affect neurons in the central nervous system through feedback regulation.

In this study, we analyzed changes in proteins and metabolites in the central nervous system of goats in both the *SS* DNA vaccine-immunized group and the negative control group. Our findings revealed significant changes in the expression of proteins associated with neuronal signal transmission, immunity, and nutrient metabolism in the hypothalamus and pituitary gland. Based on our literature survey, we identified ADCY8 as a crucial factor responsible for catalyzing the production of cAMP [17]. We observed a decrease in the expression of ADCY8, which correlated with lower levels of cAMP (Figure 3C,D). Previous studies have demonstrated that reduced cAMP levels can inhibit the secretion of hormones such as growth hormone and adrenaline. Additionally, it may also impact ion channels and reduce cell proliferation [17]. Furthermore, we noted the role of protein kinase A (PKA) in catalyzing the phosphorylation of target proteins such as the cAMP-responsive element-binding protein CREB, POU1F1, and fibronectin A. Similarly, Raf kinase catalyzes the phosphorylation of target proteins such as MAPKs. These processes play a crucial role in the regulation of growth hormone secretion by the pituitary gland in response to the *SS* vaccine [18]. In our study, we observed a significant reduction in POU1F1 expression in the pituitary gland of the *SS* vaccine-immunized group, leading to inhibited cAMP production. Conversely, we found a significant increase in STAT1 expression. The family of transcription activators (STATs) is a key contributor to GH-stimulated growth, with GH-activated STAT1, STAT3, and STAT5B promoting the transcription of the gene encoding IGF-1 [19]. These findings indicate that the effects of the *SS* vaccine on both the hypothalamus and pituitary are multiple and complex. Ultimately, the signals associated with lower SS and greater GH in the periphery are transmitted to central nervous system neurons. This dual effect allows for the continuous inhibition of cAMP production while concurrently promoting the production of IGF1 through the activation of STAT1 in the pituitary gland. As a result, the growth of animals is encouraged.

A metabolomic analysis identified DEMs resulting from *SS* DNA vaccine treatment of goats as belonging to lipid and lipid-like molecular superclass and organic acids and their derivatives. A KEGG pathway analysis revealed that *SS* DNA vaccine promotes amino acid metabolism and metabolic pathways in goats. Cholic acid is one of the important bile acids synthesized from cholesterol in the liver, whereas deoxycholic acid is a secondary bile acid produced by intestinal microorganisms under the action of bile acid hydrolase and 7α- dehydroxylase [20]. Many studies have found that good gut flora structure will benefit host health, growth, and slaughter performance. Both cholic acid and deoxycholic acid were significantly higher in abundance in the serum of *SS* DNA vaccine-immunized goats, suggesting that the *SS* DNA vaccine activated the production of deoxycholic acid by goat gut microbes. There has been an increasing number of recent studies on the association of SS and GH with gut microbes [21], but there are no reports of *SS* DNA vaccines affecting host gut microbiota. For example, a mouse model of congenital diarrheal disease has increased SS and decreased gut hormones (cholecystokinin, pancreatic dystroglycan, and neurotensin, among others), leading to lipid malabsorption and development of the gut microbes [22]. In a mouse model, the high-growth-associated microbiome altered early neuronal and oligodendrocyte development and increased circulating and brain IGF-1 levels compared with the microbiome from poorly growing preterm donors [23].

In addition, the significantly higher abundance of 2-Oxoadipic acid in the serum of SS DNA-treated goats is also of great interest. A previous study showed that 2-Oxoadipic acid is a common metabolite of tryptophan and lysine [24], suggesting that amino acid metabolism was active in the *SS* DNA vaccine-treated group of goats. In exploring the effects of dietary fiber (DF) on the gut microbiota of pigs, Wu et al. found that DF intake significantly altered the diversity of bacterial communities. Among these altered metabolites and bacterial genera, 2-Oxoadipic acid in the cecum was positively correlated with *Clostridium*, *Fibrobacterium*, and *Synechococcus*, further affecting host health, growth, and slaughter performance [25]. Notably, the multiomics results of this study highlight the significance of the sphingolipid signaling pathway, which plays a crucial role in cell membrane composition and is considered a key secondary signaling system in cells [26]. In this context, the downregulated protein ADCY8, upregulated protein KNG1, and serum sphingolipids (d18:1/18:0) have been associated with the nervous and immune systems, with studies indicating that cerebrospinal fluid levels of KNG1 can serve as a marker for cognitive deficits in Parkinson’s disease [27]. Sphingolipids (d18:1/18:0) play multiple regulatory roles in modulating immune responses, and the decrease in their abundance may be associated with stimulation from the *SS* vaccine [28]. Moreover, changes in the cGMP-PKG signaling pathway, B-cell receptors, and T-cell leukemia virus 1 infection pathway were found to be differentially enriched for proteins in the hypothalamus, suggesting that signals induced by *SS* vaccines in goats have feedback effects on the hypothalamus.

Although this study provides valuable insights into the impact of *SS* vaccines on hypothalamic and pituitary neurons, it is important to acknowledge that the findings are based solely on proteomics and metabolomic results. Furthermore, only three key DEPs were validated, and it is necessary to verify the roles of other key proteins to confirm their involvement in the growth promotion induced by the *SS* vaccine. Additionally, the signaling pathway of SS receptor-mediated pituitary GH secretion involves various protein kinases that catalyze protein phosphorylation [29]. Therefore, future studies could explore techniques such as phosphorylated proteomics to gain a more detailed understanding of complex signaling pathway networks.

## 4. Materials and Methods

### 4.1. Ethics Statement

All animal work in this study adhered to the minimum standards of animal welfare as outlined in the International Guiding Principles for Biomedical Research involving Animals (https://grants.nih.gov/grants/olaw/Guiding_Principles_2012.pdf (accessed on 23 December 2012)). The regulations of the Southwestern University Institutional Animal Care and Use Committee (IACUC-20210515-05, 15 May 2021) were followed, and all efforts were made to minimize animal suffering and improve quality of life.

### 4.2. Experimental Animals and Immunization

The animal experiment was conducted at the farm of Southwest University (Beibei, Chongqing, China). Eight 7-month-old male wethers with similar body weights were used. The wethers were equally divided into two groups: an immunized group and a negative control group. These wethers were housed under the same conditions, provided with water ad libitum, and fed twice a day at 7 a.m. and 3 p.m. with commercial full-price pellet feed obtained from Pizhou Xiaohe Technology Development Ltd. (Xuzhou, China). The nutrient levels of the commercial full-price pellet diets are presented in Table S1.

The Salmonella typhimurium-delivered SS vaccine *X9241* (ptCS/2SS-asd) was constructed early by our laboratory. The wethers in the immunized group were orally immunized with the attenuated *X9241* (ptCS/2SS-asd) at a dose of 5 × 10^9^ CFU, whereas those in the negative control group were orally immunized with the empty vector vaccine *X9241* (pVAX-asd). Prior to vaccination, each goat received 15 mL of 7.5% sodium bicarbonate solution orally to neutralize stomach acid. Booster vaccination was administered at the 2nd and 4th weeks after the initial immunization. Following the initial or booster immunization, the experimental wethers did not exhibit any signs of inflammation or disease at the injection site.

Blood samples (5 mL) were collected in duplicate from the external jugular vein of the goats (*n* = 8) at 0, 2, 4, and 8 weeks after immunization to obtain blood and serum, which were subsequently stored at −80 °C for future use. At 8 weeks after the initial immunization, three goats were randomly selected from both the negative control and experimental groups. All six selected goats were euthanized in accordance with the Southwestern University Institutional Animal Care and Use Committee (IACUC-20210515-05). The hypothalamus and pituitary gland were immediately removed, washed in cold phosphate-buffered saline (PBS), and promptly stored in liquid nitrogen.

### 4.3. Proteomic Analysis

The methods used for protein extraction and LC–MS analysis can be found in the Supplementary Materials. The database used for protein identification was uniprot_Capra_hircus_35493_20210311.fasta. Protein identification and quantitative analysis were conducted by searching the MS raw data of each sample using the MASCOT engine (Matrix Science, London, UK; version 2.2) integrated in the Proteome Discoverer 1.4 software application. The peptide mass tolerance was set at ±20 ppm, and the fragment mass tolerance was set at 0.1 Da. Proteomic analyses were performed using unpaired t tests, and heatmaps were generated using Z scores and GraphPad Prism 6.01. Additionally, principal component analysis (PCA), Kyoto Encyclopedia of the Genome (KEGG), and gene ontology (GO) were utilized, and volcano plots were created through the cloud platform of Gene Denovo Biological Co., Ltd. To identify significant DEPs, we applied the criteria of a fold change (FC) >1.2 or <0.83 with a *p* value < 0.05 for the *t* test. Hierarchical clustering, KEGG, and GO enrichment analyses were conducted on proteins that exhibited significant changes in abundance following immunization with the SS vaccine. Proteins were identified based on the criteria of having unique peptides with at least 99% confidence and a false discovery rate (FDR) ≤ 0.01.

The identified collections of differentially expressed proteins were annotated using Blast2GO for GO analysis. In addition, KEGG pathway annotation of the target protein collections was performed using the KEGG Automatic Annotation Server (KAAS) software application (https://www.genome.jp/tools/kaas/ (accessed on 1 June 2024)). For enrichment analysis, the entire set of quantified proteins was used as the background dataset, and Fisher’s exact test was used to assess enrichment. To account for multiple testing, the derived *p* values were adjusted using Benjamini–Hochberg correction. Only functional categories and pathways with *p* < 0.05 were considered statistically significant.

### 4.4. Metabolomic Analysis

The methods for metabolite extraction and LC–MS conditions are provided in the Supplemental Materials. Metabolites were identified using an in-house database established with authentic standards, and the analysis was conducted at Shanghai Applied Protein Technology Co., Ltd. (Shanghai, China), where all 239 metabolites were detected. The processed data were subjected to multivariate data analysis, including Pareto-scaled PCA and orthogonal partial least-squares discriminant analysis (OPLS-DA), using the R package (https://www.r-project.org/ (accessed on 1 June 2024)). Model robustness was evaluated using 7-fold cross-validation and response permutation testing. The variable importance in the projection (VIP) value of each variable in the OPLS-DA model was calculated to determine its contribution to the classification. Student’s t test was used to assess differences between two groups of independent samples. Pearson’s correlation analysis was performed to examine correlations between variables. For a metabolite to be considered significant, it had to meet the following criteria: VIP > 1, FC > 1.2 or <0.83, and *p* value less than 0.05. Hierarchical clustering and KEGG pathway analysis were subsequently conducted for the significantly altered metabolites.

### 4.5. Western Blotting

Methods and conditions for Western blotting are provided in the Supplementary Materials. The membranes embedded with hypothalamic protein messages were then incubated with anti-ADCY8 Rabbit pAb (1:1000; Proteintech, Wuhan, China) overnight in a refrigerator at 4 °C, and membranes containing pituitary proteins were incubated with anti-POU1F1 Rabbit pAb (1:500; ABclonal, Wuhan, China) overnight. ABclonal, Wuhan, China) and anti-STAT1 Rabbit pAb (1:2000; Proteintech, Wuhan, China) were incubated together overnight. β-actin rabbit monoclonal antibody (1:5000; Proteintech, Wuhan, China). After washing with TBST buffer, the membranes were incubated with secondary antibody against rabbit (1:2000; Proteintech, Wuhan, China) for 2 h at room temperature. After washing at least three times, protein bands were visualized by ECL (Bioground, Chongqing, China). Protein density was measured using the Image Lab 6.1 software (Bio-Rad, Hercules, CA, USA).

### 4.6. Statistical Analysis

In this experiment, an unpaired t test was performed using the GraphPad Prism 6.0 software package (San Diego, CA, USA) on all data generated in the negative control and immunized groups. The statistical data in the experiment are shown in the format of mean ± standard deviation (SD). *p* < 0.05 was considered to be statistically significant.

## 5. Conclusions

This study demonstrates that response signals in the peripheral blood and lymphatic circulation may feedback to CNS neurons after oral and nasal ingestion of *SS* vaccine in goats. The hypothalamus inhibits cAMP production by decreasing the abundance of ADCY8. STAT1 in the pituitary gland is activated, thereby promoting IGF1 production. In addition, the multiomics results of this study point to an important role for the sphingolipid signaling pathway.

## Data Availability

Data is contained within the article and Supplementary Material.

## Supplementary Materials

The following supporting information can be downloaded at: https://www.mdpi.com/article/10.3390/ijms25136888/s1.
