# Supplementary material for: Combined Proteomic and Metabolomic Analysis Reveals Comprehensive Regulation of Somatostatin DNA Vaccine in Goats
Source: Int J Mol Sci. 2024 Jun 23;25(13):6888. doi: 10.3390/ijms25136888 (PMC11241611; doi:10.3390/ijms25136888)
Supplement: Supplementary file 1 [file ijms-25-06888-s001.zip › supplementary materials.pdf]

Supplementary materials

1 Methods

1.1 Protein extraction

Hypothalamic and pituitary samples were lysed and proteins extracted using SDT (4% SDS, 100 mM Tris-HCl, 1 mM DTT, pH 7.6) buffer. The amount of protein was quantified with the BCA Protein Assay Kit (Bio-Rad, USA). Protein digestion by trypsin was performed according to filter-aided sample preparation (FASP) procedure described by Matthias Mann. The digest peptides of each sample were desalted on C18 Cartridges (Empore™ SPE Cartridges C18 (standard density), bed I.D. 7 mm, volume 3 ml, Sigma), concentrated by vacuum centrifugation and reconstituted in 40 µl of 0.1% (v/v) formic acid.

The peptide content was estimated by UV light spectral density at 280 nm using an extinctions coefficient of 1.1 of 0.1% (g/L) solution that was calculated on the basis of the frequency of tryptophan and tyrosine in vertebrate proteins. 20 µg of protein for each sample were mixed with 5X loading buffer respectively and boiled for 5 min. The proteins were separated on 12.5% SDS-PAGE gel (constant current 14 mA, 90 min). Protein bands were visualized by Coomassie Blue R-250 staining. 100 µg peptide mixture of each sample was labeled using TMT reagent according to the manufacturer's instructions (TMT 16 plex Isobaric Label Reagent (Thermo，A44520)). Each sample was separated at 300 nL/min for 90 minutes using the HPLC Liquid System Easy nLC (Thermo Fisher Scientific). From 0.00 to 3.00 minutes, the linear gradient of Buffer B was from 0% to 7%; from 3.00 to 68 minutes, the linear gradient of Buffer B was from 7% to 30%; from 68 to 83 minutes, the linear gradient of Buffer B was from 30% to 55%; from 83 to 85 minutes, the linear gradient of Buffer B was from 55% to 100%; and from 85 to 90 minutes, Buffer B was held at 100%.

1.2 LC-MS/MS analysis for peptides separation

Each sample was separated using the HPLC liquid phase system Easy nLC with nanolitre flow rate. Buffer A was 0.1% formic acid in water and B was 0.1% formic acid in acetonitrile (84% acetonitrile). The chromatographic column was equilibrated with 95% of liquid A. The samples were uploaded from the autosampler to the uploading column (Thermo Scientific Acclaim PepMap100, 100µm*2cm, nanoViper C18) through the analytical column (Thermo scientific EASY column, 10cm, ID75µm, 3µm, C18-A2) at a flow rate of 300nL/min. After chromatographic separation, the samples were analysed by mass spectrometry (MS) using a Q-Exactive mass spectrometer (Thermo Scientific).

1.3 Extraction of metabolites

The serum samples were thawed at 4 °C and mixed with 1mL of cold methanol/acetonitrile/water solution (2:2:1, v/v/v), followed by low-temperature sonication for 30 min, standing at -20 °C for 10 min, centrifugation at 14,000 g for 20 min at 4 °C, and vacuum drying of the supernatant. For mass spectrometry analysis, 100µL of acetonitrile aqueous solution (acetonitrile: water=1:1, v/v) was added, vortexed, and centrifuged at 14000 g for 15 min at 4°C, and the supernatant was taken into the sample for analysis.

1.4 UHPLC separation and Q-TOF mass spectrometry of metabolites

The samples were separated on a HILIC column of an ultra-high performance liquid chromatography (UHPLC) system (1290 Infinity LC, Agilent Technologies). The temperature of the column was 25°C, the flow rate was 0.5 mL/min and the

injection volume was 2μL. Buffer A: double-distilled water containing 25 mM ammonium acetate and 25 mM ammonia. Buffer B: acetonitrile. The samples were placed in an autosampler at 4 °C throughout the analysis. Quality control (QC) samples were placed in a sample queue to monitor and evaluate the stability of the system and the reliability of the experimental data. Primary and secondary spectra of the samples were collected using a quadrupole time-of-flight spectrometer (AB Sciex TripleTOF 6600) from Shanghai Applied Protein Technology Co.,Ltd.

1.5 Western blotting

Hypothalamic and pituitary tissue, previously stored at -80°C, was extracted and lysate (RIPA lysis buffer: protease inhibitor = 1:100) was added at a ratio of 1:10 and pulverized to homogeneity at low temperature. The resulting mixture was centrifuged at 12000 g at 4°C for 10 min. Total proteins were extracted separately and their concentration was determined using a protein concentration assay kit. Total proteins were subjected to SDS-PAGE gel electrophoresis and transferred to PVDF membrane. These membranes were blocked with 5% skimmed milk for 2 h. Membranes embedded with hypothalamic protein messages were then incubated with anti-ADCY8 Rabbit pAb (1:1000; Proteintech, Wuhan, China) overnight in a refrigerator at 4°C, and membranes containing pituitary proteins were incubated with anti-POU1F1 Rabbit pAb (1:500; ABclonal, Wuhan, China) overnight. ABclonal, Wuhan, China) and anti-STAT1 Rabbit pAb (1:2000; Proteintech, Wuhan, China) were incubated together overnight. β-actin rabbit monoclonal antibody (1:5000; Proteintech, Wuhan, China). After washing with TBST buffer, the membranes were incubated with secondary antibody against rabbit (1:2000; Proteintech, Wuhan, China) for 2 h at room temperature. After washing at least three times, protein bands were visualized by ECL (Bioground, Chongqing, China).
