# Supplementary material for: Combined Proteomic and Metabolomic Analysis Reveals Comprehensive Regulation of Somatostatin DNA Vaccine in Goats
Source: Int J Mol Sci. 2024 Jun 23;25(13):6888. doi: 10.3390/ijms25136888 (PMC11241611; doi:10.3390/ijms25136888)
Supplement: Supplementary file 1 [file ijms-25-06888-s001.zip › supplementary tables.pdf]

Supplementary tables

Table S1. Nutrient levels (dry matter basis) for commercial full-price pellets.

Nutrient level	Immunized group	Negative control group
Digestible energy (DE), MJ/kg	12.14	12.14
Crude protein (CP),%	16.63	16.63
Crude fat (CF) ,%	2.04	2.04
Neutral detergent fiber (NDF) ,%	37.91	37.91
Acid detergent fiber (ADF),%	24.80	24.80
Crude ash (Ash),%	10.31	10.31
Calcium (Ca),%	1.08	1.08
Phosphorus (P),%	0.64	0.64

CP, CF, NDF, ADF, Ash, Ca, P were analyzed values, and DE is a calculated value.

Table S2. Details of significantly differentially expressed metabolites.

Number	Name	Ion mode	VIP	Fold change	p-value
1	Sphingomyelin (d18:1/18:0)	POS	3.5281	0.7415	0.0415
2	Cholic acid	POS	2.4838	3.8576	0.0122
3	D(-)-beta-hydroxy butyric acid	NEG	4.4150	1.4530	0.0273
4	Deoxycholic acid	NEG	1.1957	2.1478	0.0037
5	Methylmalonic acid	NEG	1.3067	0.5742	0.0232
6	Enterostatin human	NEG	1.1215	0.6438	0.0177
7	Cholic acid	NEG	3.9252	4.0631	0.0238
8	2-Oxoadipic acid	NEG	34.1491	0.7714	0.0012
